# Asymptotic consensus of hybrid multi-agent systems considering network attacks

**DOI:** 10.1038/s41598-025-17025-x

**Published:** 2025-08-23

**Authors:** Dongwei Wang, Ying Zhang

**Affiliations:** 1 Logistics Engineering College, Shanghai Maritime University, Shanghai, 201306 China; 2 College of Information Engineering, Shanghai Maritime University, Shanghai, 201306 China; 3 School of Mechanical Engineering, Shanghai Dianji University, Shanghai, 201306 China

**Keywords:** Asymptotic consensus, Hybrid multi-agent systems, Continuous time, Discrete time, Byzantine nodes, Deep dynamic heterogeneous redundancy, Ocean sciences, Engineering

## Abstract

This paper focuses on the problem of active defense and achieving asymptotic consensus control in hybrid multi-agent systems under network attacks. Considering the influence of Byzantine nodes, on the basis of the theory of mimic defense, a deep dynamic heterogeneous redundancy architecture for hybrid multi-agent systems is established. Drawing on the Lyapunov asymptotic stability theory, a hybrid multi-agent asymptotic consensus is proposed. Furthermore, a hybrid multi-agent progressive consensus clustering review strategy is suggested. Via Lyapunov asymptotic stability theory, the sufficient and necessary conditions for achieving the asymptotic consensus of hybrid multi-agent systems on the basis of a hybrid clustering review strategy are proven. The strategy’s extended application method in asymptotic proportional consensus, enabling multi-agent systems with various target tasks to achieve asymptotic proportional consensus, is presented. The theoretical results are validated through numerical examples.

## Introduction

With the rapid development of information technology, research on hybrid multi-agent systems cooperation and network security technology has attracted attention from various countries. In particular, the concepts of Maritime Autonomous Surface Ships (MASS) and multi-agent collaboration at sea are receiving increasing attention from countries. Many countries have prioritized research on MASS projects^1^, including South Korea with its self-propelled surface vessels^2^, Japan with its MEGURI 2040 projects^3^, and the EU with its Autoship projects^4^. In 2021, the Belgian company Seafar’s container ship Deseo has achieved comprehensive remote intelligent control from Zeebrugge Port to Antwerp Port^5^. In 2022, the MEGURI 2040 project in Japan conducted long-distance autonomous operation trials using ferries, container ships, and amphibious vessels, achieving multi-agent collaboration tasks^3^. While network connectivity facilitates multi-agent systems, it also increases the risk of network attacks on multi-agent systems^6^. Network attacks mainly consider denial of service (DoS) attacks that destroy data availability and false data injection (FDI) attacks that damage data integrity, and these network attacks result in the presence of Byzantine nodes in the system. Various types of network attacks can penetrate from the information layer to the physical layer and cause serious damage to physical processes, which is highly likely to cause adverse consequences such as system downtime, environmental damage, and even personnel disability. Therefore, studying the issue of state consensus in multi-agent systems under network attacks is of great practical significance for improving the stability of multi-agent systems and enhancing their security protection.

In recent years, the issues of consensus and security collaboration in multi-agent systems, especially the presence of Byzantine nodes in the system, have attracted increasing attention from researchers. Xin et al. implemented the consensus control problem for multi-agent systems on the basis of the the consensus theory of multi-agent systems^7^. An et al. proposed a Byzantine fault-tolerant algorithm based on node weights, whose decentralized mechanism enables sub leaders and leaders to work together, thereby improving transmission rates and achieving multi-agent consensus control^8^. Zheng et al. proposed three state consensus protocols for first-order hybrid multi-agent systems and proved the sufficient and necessary conditions for achieving state consensus^9^. In 2019, scholars from Xi’an University established consensus in system interaction based on game theory methods for discrete-time and continuous time systems^10^. Zeng et al. established a credit based elastic control protocol for Byzantine nodes in networks with and without leaders^11^. Shang et al. used the weighted average subsequence simplification algorithm (W-MSR) for Byzantine nodes in discrete-time and continuous time systems to achieve unified consensus in multi-agent systems^12^. Gong et al. proposed a Twin-Layer approach to solve the problem of designing countermeasures for distributed elastic output time-varying formation tracking (TVFT) in heterogeneous multi-agent systems (MASs) against general Byzantine attacks (GBAs)^13^. In addition, for network control systems with Byzantine nodes, the important role of the W-MSR algorithm in achieving consensus of network system states is explained^14–19^. For hybrid multi-agent systems, literature^20–23^ explains the related algorithms and applications of hybrid multi-agent consistency. In view of asymptotic consensus, literature^24–27^ describes the relevant theories of asymptotic consensus and its importance in multi-agent systems. For mimic defense, literature^28–30^ describes relevant theoretical algorithms of mimic defense and its importance in industrial control system security research. Pei proposed a class of distributed control protocols to achieve group consensus of multi-agent systems^31^. Huang researched how to improve secure wireless communication under multi-user and multi-eavesdropper scenarios^32^. Literatures^33–35^ studied formation-surrounding control for multi-agent under network attacks. The aforementioned works have to some extent made prior assumptions about multi-agent network signals, such as time invariance, distribution characteristics, attack signal boundaries, and multi-agent cooperation. In addressing Byzantine node issues, the approach is one of passive defense control. There is limited research on active defense and asymptotic consensus in hybrid multi-agent systems, encompassing both discrete and continuous time.

This study aims to investigate the problem of active defense and achieving asymptotic consensus control in a hybrid multi-agent system with Byzantine nodes. The main contributions of this study are presented below.

(1) On the basis of the theory of mimic defense, a deep dynamic heterogeneous redundancy architecture (DDHR) for hybrid multi-agent systems is established. Compared to the dynamic heterogeneous redundancy architecture described in reference^28^, the architecture proposed in this paper introduces a forewarn strategy module based on asymptotic consistency. This strategy deeply simulates the state change process of each agent in the computation execution set and can provide early warning of possible Byzantine nodes.

(2) On the basis of the concept of Lyapunov asymptotic stability, a hybrid multi-agent asymptotic consensus is further developed. Compared to references^12,16^, this paper defines absolute error *ε* instead of absolute error 0, which improves the computational efficiency of the system and enhances its robustness.

(3) A clustering review strategy based on hybrid multi-agent asymptotic consensus is proposed. Compared to the W-MSR algorithm in references^12,14,16^, the strategy proposed in this paper is based on the K-means algorithm principle. And the strategy can detect potential Byzantine nodes in advance with an additional round of checks within the neighborhood of each regular agent.

(4) Compared to reference^16^, based on the Lyapunov asymptotic stability theory and the aforementioned hybrid clustering review strategy, this paper not only proves the sufficient conditions for achieving asymptotic consistency in hybrid multi-agent systems, but also further proves their necessary conditions.

The remainder of this article is organized as follows. Section II introduces relevant theoretical methods and elaborates on the model and algorithm of this article in detail. On this basis, relevant conclusion proofs are provided. Section III presents an experimental comparison, and Section IV concludes the article.

## The proposed method

### Theoretical basis

Assuming that a multi-agent system contains N agents, the cooperative node is the one that has not been attacked by the network, and the Byzantine node is the one that has been attacked by the network. The communication topology between multiple agents is a directed graph with n nodes: let $G=(\upsilon ,{\varepsilon _{r(t)}},{H_{r(t)}})$ represent the directed graph with n nodes, where $\nu =\{ \upsilon {}_{1},\upsilon {}_{2},\upsilon {}_{3}, \ldots \upsilon {}_{n}\}$ is the node set, ${\varepsilon _{r(t)}}=\nu \times \nu$ is the network topology edge set. The adjacency matrix between intelligent agents is denoted as ${H_{r(t)}}$, ${H_{r(t)}}=[h_{{ij}}^{{r(t)}}] \in {R^{N \times N}}$, Where $h_{{ij}}^{{r(t)}}$ is the adjacency communication value between the th intelligent agent $i,j$. The edge nodes of the graph *G* start from ${\upsilon _i}$ and end at ${\upsilon _j}$, denoted by $({\upsilon _i},{\upsilon _j}) \in {\varepsilon _{r(t)}}$. The set of adjacent nodes formed by all the edges leading to ${\upsilon _i}$ is represented as ${{\rm N}_i}=\{ {\upsilon _j} \in \nu :({\upsilon _j},{\upsilon _i}) \in {\varepsilon _{r(t)}}\}$.

#### Lemma 1

^14^(r-reachable set): *Given a directed graph G with n nodes* $\nu =\{ \upsilon {}_{1},\upsilon {}_{2},\upsilon {}_{3}, \cdots \upsilon {}_{n}\}$ *and a non-empty S*, $\exists S \in \nu$ *, if* ${\upsilon _i} \in S$ *satisfies that* $\left| {{\upsilon _i}\backslash S} \right| \geqslant r$ *, where* $r \in Z \geqslant 0$ *, then S is said to be an* *r-reachable set.*

#### Lemma 2

^14^ (r-robustness) : *A directed graph G contains n nodes* $\nu =\{ \upsilon {}_{1},\upsilon {}_{2},\upsilon {}_{3}, \cdots \upsilon {}_{n}\}$, ($n \geqslant 2$ *), for any pair of non-empty and mutually exclusive subsets, if there is a subset that is r-reachable, then the directed graph* *G is* *r-robust.*

### Security cooperative control architecture model of hybrid multi-agent system

**Fig. 1:**
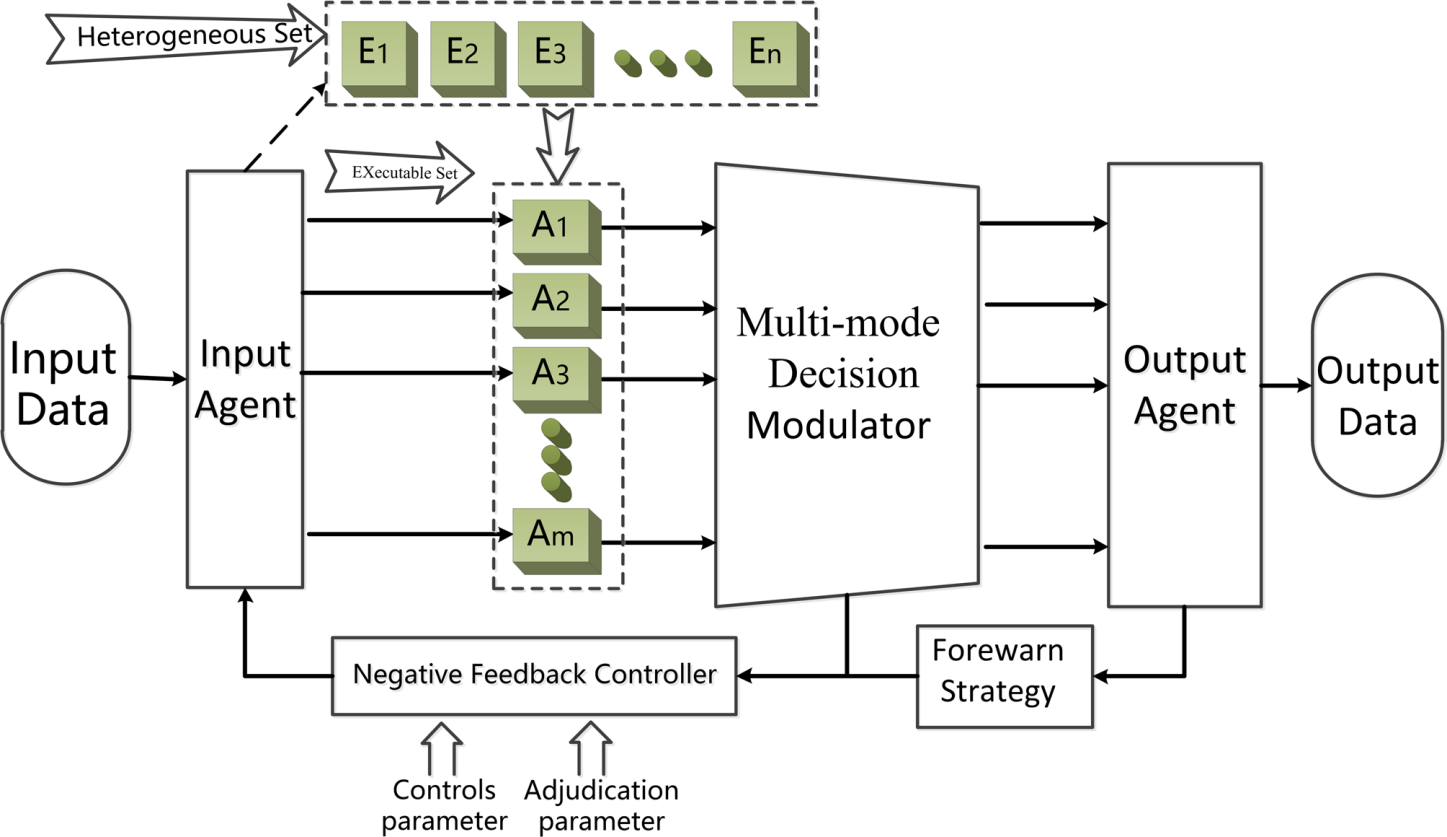
Deep dynamic heterogeneous redundancy (DDHR) architecture.

As the key to the reliable operation of hybrid multi-agent system, the importance of security protection is self-evident. Therefore, based on the idea of mimic defense, this paper proposes an improved DHR structure, namely, Deep Dynamic Heterogeneous Redundancy (DDHR) architecture, as shown in Fig. 1. ${E_1},{E_2},{E_3}, \ldots ,{E_n}$ is an heterogeneous body set *E*, and ${A_1},{A_2},{A_3}, \ldots ,{A_n}$ is an executable body set *A*.

The DDHR architecture is composed mainly of a reconfigurable execution body set, input and output agents, a negative feedback controller, multi-mode arbiter and a forewarn strategy. Based on DHR architecture, this architecture introduces a forewarn strategy module based on asymptotic consensus. Among them, the input agent module is used to receive the commands issued by the negative feedback controller and distribute the input sequence to multiple policies or randomly determined functionally-equivalent reconfigurable execution sets. The reconfigurable executive body set can independently complete the function events given by the system. The multi-mode decision modulator decides the legality of the multi-mode output vector according to the decision algorithm and selects the output data that conform to the policy to the output agent. The forewarn strategy module deeply simulates and calculates the current state of the execution set, which is mainly based on the current state and trend of state changes of the execution set. Based on asymptotic consensus, this strategy first deeply simulates the state change process of each intelligent agent in the execution agent set, and provides early warning of possible Byzantine nodes. Second, if unexpected arbitration results or warning signals occur, then the negative feedback controller is activated. After the negative feedback controller is activated, it will perform operations such as recombination, reconstruction, and reconfiguration of some actuators based on functional equivalent components. Finally, when the non compliant state of the ruling disappears or the frequency of the situation occurs within a certain threshold, the feedback control process ends and the system maintains stability.

### Hybrid multi-agent asymptotic consensus

Referring to the information-physical fusion systems^19^, it investigates a discrete linear multi-agent system under attack. The state expression of the i-th intelligent agent is as follows.

1 $$\left\{ {\begin{array}{*{20}{l}} {{x_i}(k+1)=A{x_i}(k)+B{u_i}(k)+{\delta _i}(k)} \\ {{y_i}(k)=E{x_i}(k)+{\eta _i}(k)} \end{array}} \right.$$

where, ${x_i}(k) \in {R^n}$ is the state of the intelligent agent system *i* at time *k*; ${u_i}(k) \in {R^m}$ is the control input signal of the intelligent agent system at time *k*; ${\delta _i}(k) \in {R^n}$ represents Gaussian white noise at time *k*; ${y_i}(k) \in {R^p}$ is the potential output signal from Byzantine nodes at time *k*; ${\eta _i}(k) \in {R^p}$ is the tampering signal generated by Byzantine nodes at time *k*; and $A \in {R^{n \times n}},B \in {R^{n \times m}},E \in {R^{p \times n}}$ are known system parameters with appropriate dimensions.

Drawing on the continuous system with malicious attacks^12^, the expression for the *i*-th intelligent agent is as follows.

2 $$\left\{ {\begin{array}{*{20}{l}} {{{\dot {x}}_i}(t)=A^{\prime}{x_i}(t)+B^{\prime}{u_i}(t)+{\delta _i}(t)} \\ {{y_i}(k)=E^{\prime}{x_i}(t)+{\eta _i}(t)} \end{array}} \right.$$

where, ${x_i}(t) \in {R^n}$ is the system state of the i-th intelligent agent system; ${u_i}(t) \in {R^m}$ is the control law of the i-th intelligent agent system; ${y_i}(t) \in {R^p}$ is the potential output signal from Byzantine nodes for the i-th intelligent agent system; ${\delta _i}(t) \in {R^n}$ represents Gaussian white noise for the i-th intelligent agent system; ${\eta _i}(t) \in {R^p}$ is the tampering signal generated by Byzantine nodes for the i-th intelligent agent system; and $A^{\prime} \in {R^{n \times n}},\;B^{\prime} \in {R^{n \times m}},\;E^{\prime} \in {R^{p \times n}}$ are known system parameters with appropriate dimensions.

Since hybrid multi-agent systems include discrete-time and continuous-time systems, based on the above models, let ${\nu ^C}=\left\{ {{\upsilon _1},{\upsilon _2}, \ldots ,{\upsilon _m}} \right\}$ represent the continuous-time system, ${\nu ^D}=\left\{ {{\upsilon _{m+1}},{\upsilon _{m+2}}, \ldots ,{\upsilon _n}} \right\}$ represent the discrete-time system, *B* denotes Byzantine nodes, and the hybrid multi-agent system model is established as presented below.

**Fig. 2:**
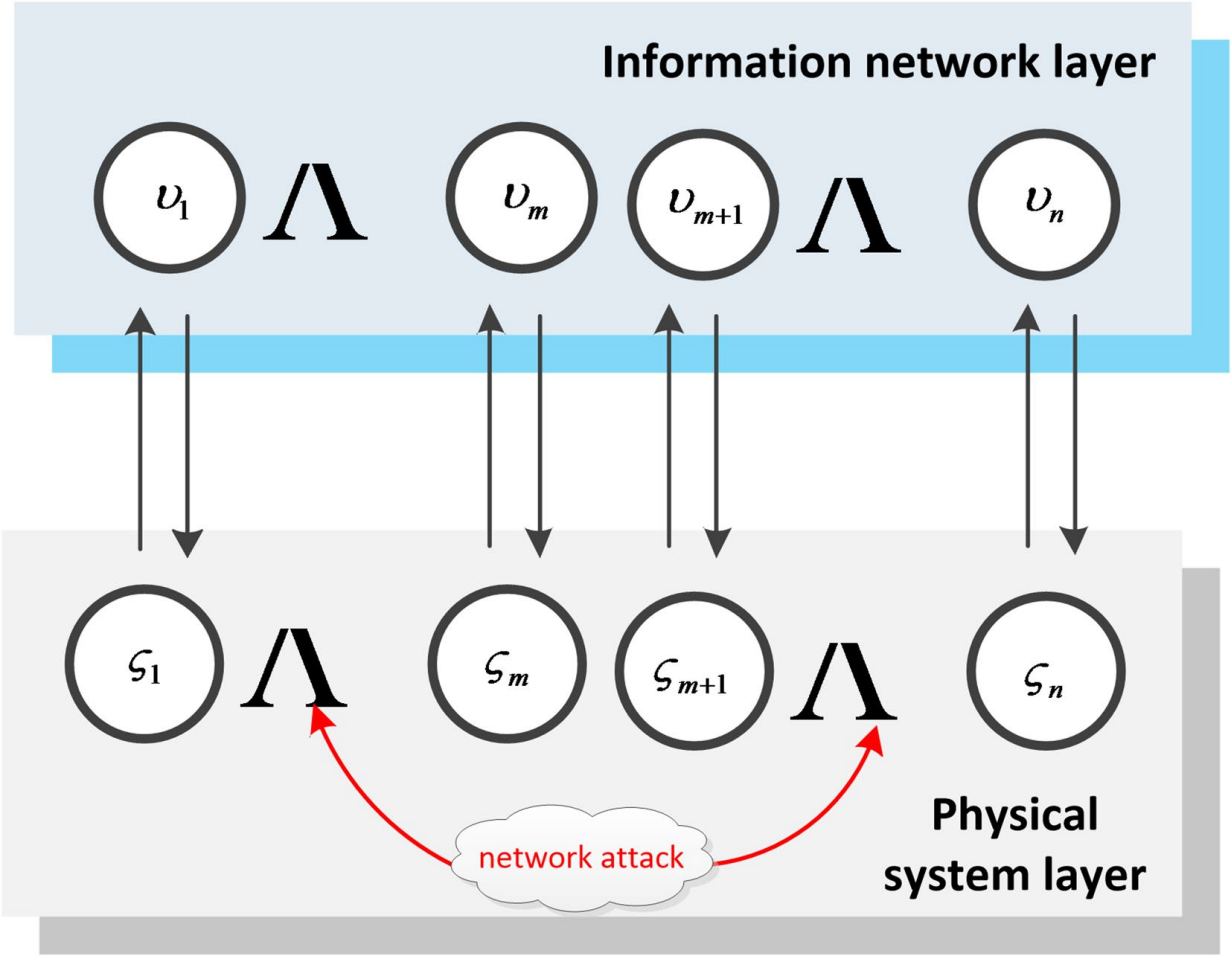
Hybrid multi-agent system model.

Since hybrid multi-agent systems include discrete-time and continuous-time systems, based on the above models, a hybrid multi-agent system model is established as shown in Fig. 2, where, ${\varsigma _1}, \ldots ,{\varsigma _m}$ represents the intelligent agents of m continuous time systems in the physical system layer, ${\upsilon _1}, \ldots ,{\upsilon _m}$ represents the m continuous time systems mapped by the physical system layer in the information network layer, and let ${\nu ^C}=\left\{ {{\upsilon _1},{\upsilon _2}, \ldots ,{\upsilon _m}} \right\}$ represent the set of continuous time systems. Similarly, ${\varsigma _{m+1}}, \ldots ,{\varsigma _n}$ represents the intelligent agents of the n-m discrete-time systems in the physical system layer, ${\upsilon _{m+1}}, \ldots ,{\upsilon _n}$ is the mapping of the n-m discrete-time systems in the information network layer of the physical system layer, and ${\nu ^D}=\left\{ {{\upsilon _{m+1}},{\upsilon _{m+2}}, \ldots ,{\upsilon _n}} \right\}$ represents the set of discrete-time systems. When there is a network attack, *B* represents Byzantine nodes, and *C* represents cooperative nodes that have not been attacked by the network. When $t \geqslant 0$, ${x_i}(t) \in R$ (or ${x_i}(k) \in R$) is used to represent continuous- time dynamics (or discrete-time dynamics).

The formula (1) describes the attacked multi-agent discrete system, whereas formula (2) describes the attacked multi-agent continuous system. Then, in the hybrid multi-agent system, the state of the i-th multi-agent at time t can be described as follows:

For the nodes of continuous-time dynamic systems: ${\upsilon _i} \in C \cap {\nu ^C},$

3 $${\dot {x}_i}(t)={\gamma _i}^{C}\left( {\left\{ {x_{j}^{i}\left( t \right):{\upsilon _j} \in \left( {{N_i} \cup \left\{ {{\upsilon _i}} \right\}} \right) \cap {\nu ^C}} \right\}} \right) \cup \left\{ {x_{j}^{i}\left( k \right):{\upsilon _j} \in {N_i} \cap {\nu ^D}} \right\}$$

where, $$k \in Z,$$ $$t \in [k,k+1).$$

And for the nodes of discrete-time dynamic systems, ${\upsilon _i} \in C \cap {\nu ^D},$

4 $${x_i}(k+1)=\gamma _{i}^{D}\left( {\left\{ {x_{j}^{i}\left( t \right):{\upsilon _j} \in \left\{ {{N_i} \cup {\upsilon _i}} \right\}} \right\}} \right)$$

where the function $\gamma _{i}^{C}\left( \cdot \right)$ represents the change states for the continuous-time system, ${u_i}(t)$ in Formula (2) follows the rules of function $\gamma _{i}^{C}\left( \cdot \right)$ and $\gamma _{i}^{D}\left( \cdot \right)$ represents the change states for discrete-time system, ${u_i}(k)$ in Formula (1) follows the rules of function $\gamma _{i}^{D}\left( \cdot \right)$. $x_{j}^{i}\left( t \right)$ represents the state transmitted from node j to node i at time t, where $x_{j}^{i}\left( t \right)=x_{j}^{{}}\left( t \right)$, and ${\upsilon _j} \in C$. For discrete-time dynamic systems, when $t \in [k,k+1)$, note that $x_{i}^{{}}\left( t \right)=x_{i}^{{}}\left( k \right)$, i.e., within the interval $t \in [k,k+1)$, the information transmitted from neighboring nodes of ${\upsilon _i} \in {\nu ^D}$ to the cooperative node remains unchanged.

In accordance with the Lyapunov function asymptotic stability theorem, the asymptotic consensus of hybrid multi-agent systems is expounded for the research content of this paper.

#### Definition 1

(Asymptotic consensus of Hybrid Multi-Agent Systems): In a hybrid multi-agent system, for any initial state of the nodes, as time progresses, all cooperative nodes can reach a consistent steady state. This situation is termed as asymptotic consensus in the hybrid multi-agent system. That is: $\forall {\left\{ {x_{i}^{{}}\left( 0 \right)} \right\}_{{\upsilon _i} \in \nu }}$, $\exists \varepsilon \in R$, $\forall {\upsilon _i},{\upsilon _j} \in C \cap {\nu ^C}$, ${\lim _{t \to \infty }}\left| {{x_i}\left( t \right) - {x_j}\left( t \right)} \right| \leqslant \varepsilon$ and ${\lim _{t \to \infty }}\left| {{x_i}\left( {t+\Delta t} \right) - {x_i}\left( t \right)} \right| \leqslant \varepsilon$. Additionally, where $\forall {\upsilon _i},{\upsilon _j} \in C$, ${\lim _{k \to \infty }}\left| {{x_i}\left( k \right) - {x_j}\left( k \right)} \right| \leqslant \varepsilon$ and ${\lim _{k \to \infty }}\left| {{x_i}\left( {k+1} \right) - {x_i}\left( k \right)} \right| \leqslant \varepsilon$, then it is said that the cooperative nodes ${\upsilon _i},{\upsilon _j} \in C$ of the hybrid multi-agent system achieve asymptotic consensus.

In the study of asymptotic consensus in hybrid multi-agent systems, the description of Byzantine nodes in the context of network attacks is provided as follows:

#### Byzantine nodes ^14^

A node, ${\upsilon _i}$, is considered a Byzantine node if it sends inconsistent information or does not follow the system’s designated policies, or sends other information that disrupts system functionality to its neighboring nodes at the same time, i.e., ${\upsilon _i} \in {\text{B}}$.

Owing to the capability of Byzantine nodes to update and transmit state information, whether the network system operates in a broadcast or point-to-point manner, it will have a detrimental impact on the system. If the intelligent agent under network attack becomes the dominant player of the system, then the system will be unable to achieve consensus. These Byzantine nodes do not comply with system policies and disrupt system operation. Therefore, it is necessary to design reasonable algorithms to reduce or eliminate the influence of Byzantine nodes without knowing the identities of each multi-agent node.

### Cluster review strategy

In the hybrid multi-agent secure collaborative control architecture, the proposed a forewarn strategy is to deeply simulate and calculate the state change process of each agent in the execution agent set. This is achieved through a clustering review strategy based on asymptotic consensus to determine whether each intelligent agent has reached asymptotic consensus. Consequently, this strategy can provide early warning of possible Byzantine nodes. The specific details of the clustering review strategy are presented below.

A hybrid multi-agent system comprises *N* nodes. Based on the principles of the K-means clustering algorithm, at any given moment *k*, each node ${\upsilon _i}$ receives information sent from neighboring nodes. These neighboring nodes might exhibit Byzantine behaviors, but node ${\upsilon _i}$ cannot ascertain which nodes might have been compromised. To enable node ${\upsilon _i}$ to update its state securely, an asymptotic consensus clustering review strategy for the hybrid multi-agent system is designed. This strategy eliminates Byzantine nodes from the neighboring nodes, ensuring the system’s asymptotic consensus. This strategy is termed the Hybrid Clustering Review Strategy. Taking K = 1 as an example, this clustering review strategy is described as follows:

1) Form an information list.

At any given moment, *k*, each node, ${\upsilon _i}$, receives information from neighboring nodes and forms an information list, namely, $\forall k \in Z$, ${\upsilon _i} \in {\nu ^C}$. At moment $t \in [k,k+1)$, the information from neighboring nodes is received $\left\{ {x_{j}^{i}\left( t \right)} \right\}$, (where ${\upsilon _j} \in {\nu ^D}$, $x_{j}^{i}\left( t \right)=x_{j}^{i}\left( k \right)$), and forms an information list $\left\{ {x_{1}^{i}\left( t \right),\;x_{2}^{i}\left( t \right), \ldots x_{m}^{i}\left( t \right),\;x_{{m+1}}^{i}\left( t \right), \ldots x_{{\text{n}}}^{i}\left( t \right)} \right\}$.

2) Compute the clustering center.

Compute the clustering center ${x^{\prime}_i}$ of the information list $\left\{ {x_{j}^{i}\left( t \right)} \right\}$:

5 $${x^{\prime}_i}\left( t \right)=\frac{1}{{\left| {\left\{ {x_{j}^{i}\left( t \right)} \right\}} \right|}}\sum\limits_{{j \in {\nu ^C} \cup {\nu ^D}}} {x_{j}^{i}\left( t \right)}$$

3) Judge the ment node attribute.

Set an appropriate threshold, $\theta ^{\prime}$. For at least one node, ${\upsilon _i}$, if $\left| {x_{j}^{i}\left( t \right) - {{x^{\prime}}_i}\left( t \right)} \right| \leqslant \theta ^{\prime}$ holds true, then this node is considered as a cooperative node. If $\left| {x_{j}^{i}\left( t \right) - {{x^{\prime}}_i}\left( t \right)} \right|>\theta ^{\prime}$ holds true, then this node is considered not meeting the requirements, and set its weight to 0.

4) Update the node status.

Continuously update the node states and verify whether hybrid multi-agent asymptotic consensus is achieved, i.e., whether ${\lim _{t \to \infty }}{x_i}\left( t \right) - {x_j}\left( t \right) \leqslant \varepsilon$ and ${\lim _{k \to \infty }}{x_i}\left( k \right) - {x_j}\left( k \right) \leqslant \varepsilon$. If it is satisfied, then proceed to step 5). If not satisfied, then return to step 2) for recalculation. Exit the loop and proceed to step 5) when the loop limit is reached.

5) Obtain the output result.

Nodes that meet asymptotic consensus criteria are designated as cooperative nodes and nodes that do not meet the criteria are identified as Byzantine nodes, removing them. The set of removed nodes is represented by the set ${R_i}(t)$.

In a hybrid multi-agent system, nodes synchronize and interact with each other through the mutual exchange of information. Each node updates its own state according to specified rules, modeled as follows: for the nodes of a continuous-time dynamic system ${\upsilon _i} \in C \cap {\nu ^C}$, $k \in Z$, using the rule function $\gamma _{i}^{C}\left( \cdot \right)$ in formula (3) obtains the following:.

6 $${\dot {x}_i}(t)=\sum\limits_{{{\upsilon _j}}} {{\gamma _{ij}}\left( {x_{j}^{i}\left( t \right),{x_i}\left( t \right)} \right)} +\sum\limits_{{{\upsilon _j}}} {{\gamma _{ij}}\left( {x_{j}^{i}\left( k \right),{x_i}\left( t \right)} \right)}$$

For the continuous-time system function ${\upsilon _j} \in \left[ {\left( {{N_i} \cup \left\{ {{\upsilon _i}} \right\}} \right)\backslash {{\text{R}}_i}(t)} \right] \cap {\nu ^C}$ and the discrete-time system functions ${\upsilon _j} \in \left[ {{N_i}\backslash {{\text{R}}_i}(t)} \right] \cap {\nu ^D}$ and $t \in [k,k+1)$, the following conditions should be met:

(1) The rule function ${\gamma _{ij}}$ is locally Lipschitz continuous,

(2) $${\gamma _{ij}}\left( {x_{j}^{{}}\left( t \right),x_{i}^{{}}\left( t \right)} \right)=0 \Leftrightarrow x_{j}^{{}}\left( t \right)=x_{i}^{{}}\left( t \right)$$,

(3) $${\gamma _{ij}}\left( {x_{j}^{{}}\left( t \right),x_{i}^{{}}\left( t \right)} \right)\left( {x_{j}^{{}}\left( t \right) - x_{i}^{{}}\left( t \right)} \right)>0$$, $${x_j}\left( t \right) \ne {x_i}\left( t \right)$$.

When the aforementioned hybrid multi-agent system consensus algorithm is applied, each node, ${\upsilon _i}$, receives information sent by neighboring nodes and forms an information list. Next, it calculates the clustering center ${x^{\prime}_i}$ of the information list $\left\{ {x_{j}^{i}\left( t \right)} \right\}$. Then by setting an appropriate threshold, it assigns a weight of 0 to nodes that do not meet the threshold requirements. It continuously updates the current status of nodes, ultimately identifying and eliminating Byzantine nodes.

For nodes in a discrete-time dynamic system ${\upsilon _i} \in C \cap {\nu ^D}$, the rule function ${\gamma _i}^{D}\left( \cdot \right)$ in formula (4) is utilized as follows:

7 $${x_i}(k+1)=\sum\limits_{{{\upsilon _j}}} {{\omega _{ij}}} (k)x_{j}^{i}(k)$$

where, ${\upsilon _j} \in \left( {{N_i} \cup \left\{ {{\upsilon _i}} \right\}} \right)\backslash {{\text{R}}_i}(k)$, $\forall ({\upsilon _j},{\upsilon _i}) \in {\varepsilon _{r(t)}}$, and ${\omega _{ij}}(k)$ represent non-negative weights, satisfying:

(1) If$${\upsilon _j} \notin \left( {{N_i} \cup \left\{ {{\upsilon _i}} \right\}} \right)$$, then $${\omega _{ij}}(k)=0$$.

2 $$\sum\limits_{{{\upsilon _j} \in \left( {{N_i} \cup \left\{ {{\upsilon _i}} \right\}} \right)\backslash {{\text{R}}_i}(k)}} {{\omega _{ij}}} (k)=1$$

#### Note 1

For discrete-time dynamic systems (7), typically ${\omega _{ij}}(k)={\left( {\left| {{N_i}} \right|+1 - \left| {{R_i}(k)} \right|} \right)^{ - 1}}$, for continuous-time dynamic systems (6), ${\gamma _{ij}}\left( {x_{j}^{{}}\left( t \right),x_{i}^{{}}\left( t \right)} \right)={\alpha _{ij}}\left( {x_{j}^{{}}\left( t \right) - x_{i}^{{}}\left( t \right)} \right)$, and ${\alpha _{ij}}>0$ are the weights of the system network. The algorithm described above is referred to as the hybrid multi-agent system asymptotic consensus algorithm.

### Main results and theoretical analysis

To achieve secure and rapid communication among hybrid multi-agent systems, this section primarily investigates the consensus of the hybrid multi-agent system (1)-(7) under network attacks using the S-local bounded model. Initially, for all cooperative nodes, the maximum and minimum state values among all cooperative nodes are expressed via $M(t)$ and $m(t)$, respectively. Specifically, $t \geqslant 0$, $M(t)={\hbox{max} _{{\upsilon _i} \in {\text{C}}}}x(i)$, and $m(t)={\hbox{min} _{{\upsilon _i} \in C}}x(i)$. This approach is applicable to both continuous-time and discrete-time systems.

#### Theorem 1

A multi-agent system,$G=(\upsilon ,{\varepsilon _{r(t)}},{H_{r(t)}})$, adopts the aforementioned hybrid clustering review strategy within an S-local bounded model containing S Byzantine nodes. According to the principles of the S-local bounded model, when $t \geqslant 0$, and ${\upsilon _i} \in C \cap {\nu ^C}$, then ${x_i}(t) \in \left[ {m(0),M(0)} \right]$, and when $k \in Z$, and ${\upsilon _i} \in {\text{C}} \cap {\nu ^D}$, then ${x_i}(k+1) \in \left[ {m(k),M(k)} \right]$.

#### Proof

When $k \in Z$, ${\upsilon _i} \in C \cap {\nu ^D}$, according to the S-local bounded model and the adoption of the aforementioned hybrid clustering review strategy, in Formula (7), ${x_i}(k+1)$ is a convex combination, therefore ${x_i}(k+1) \in \left[ {m(k),M(k)} \right]$.

When $t \geqslant 0$, ${\upsilon _i} \in {\text{C}} \cap {\nu ^C}$, it is only necessary to prove that ${x_i}(t) \leqslant M(0)$, and similarly, ${x_i}(t) \geqslant m(0)$ can be proven. Assuming that when $t \geqslant 0$, and ${\upsilon _i} \in C \cap {\nu ^C}$, there exists ${x_i}(t)>M(0)$, then there exist ${t^ * } \leqslant t,\;{k^ * } \in Z$, ${t^ * } \in [{k^ * },{k^ * }+1)$, satisfying: (1) $\forall t^{\prime} \leqslant {t^ * }$, ${x_j}(t^{\prime}) \leqslant M(0)$, and (2) ${\dot {x}_i}({t^ * })>0$, ${x_i}({t^ * })=M(0)$. According to Formula (6):

8 $${\dot {x}_i}(t)=\sum\limits_{{{\upsilon _j}}} {{\gamma _{ij}}\left( {x_{j}^{i}\left( t \right),x_{i}^{{}}\left( t \right)} \right)} +\sum\limits_{{{\upsilon _j}}} {{\gamma _{ij}}\left( {x_{j}^{i}\left( k \right),\;x_{i}^{{}}\left( t \right)} \right)}>0$$

As it is not possible to have S + 1 Byzantine nodes in the S-local bounded model, adopting the hybrid clustering review strategy as described above, we have that $\forall {\upsilon _j} \in \left[ {\left( {{N_i} \cup \left\{ {{\upsilon _i}} \right\}} \right)\backslash {{\text{R}}_i}(t)} \right] \cap {\nu ^C}$, and $x_{j}^{i}\left( t \right) \leqslant x_{i}^{{}}\left( {{t^ * }} \right)=M(0)$. Simultaneously, $\forall {\upsilon _j} \in \left[ {{N_i}\backslash {{\text{R}}_i}(t)} \right] \cap {\nu ^D}$, and $x_{j}^{i}\left( {{k^ * }} \right) \leqslant x_{i}^{{}}\left( {{t^ * }} \right)=M(0)$. According to the rules 2) and 3) of the function ${\gamma _{ij}}$, then ${\dot {x}_i}(t)=\sum\limits_{{{\upsilon _j}}} {{\gamma _{ij}}\left( {x_{j}^{i}\left( t \right),x_{i}^{{}}\left( t \right)} \right)} +\sum\limits_{{{\upsilon _j}}} {{\gamma _{ij}}\left( {x_{j}^{i}\left( k \right),\;x_{i}^{{}}\left( t \right)} \right)} =0$. This finding contradicts the computation in Formula (8). Therefore, the assumption is not valid. When $t \geqslant 0$, ${\upsilon _i} \in {\text{C}} \cap {\nu ^C}$, and ${x_i}(t) \leqslant M(0)$, and similarly, ${x_i}(t) \geqslant m(0)$.

#### Theorem 2

If a multi-agent system $G=(\upsilon ,{\varepsilon _{r(t)}},{H_{r(t)}})$ adopts the aforementioned hybrid clustering review strategy within an S-local bounded model containing S Byzantine nodes, according to the principles of the S-local bounded model, the sufficient condition for the system’s asymptotic consensus is as follows: $G=(\upsilon ,{\varepsilon _{r(t)}},{H_{r(t)}})$ is at least (2 S + 1)-robustness, and the necessary condition is as follows: $G=(\upsilon ,{\varepsilon _{r(t)}},{H_{r(t)}})$is at least (S + 1)-robustness.

#### (Necessary Condition) proof

If the system *G* does not achieve (S + 1)-robustness, then there exist two non-empty, mutually exclusive subsets that do not satisfy (S + 1)-reachable sets. In other words, each node in these two non-empty, mutually exclusive subsets has only S neighboring nodes outside of these subsets. If the nodes that satisfy $\left| {\left\{ {x_{j}^{i}\left( t \right)} \right\} - {{x^{\prime}}_i}\left( t \right)} \right| \leqslant \theta ^{\prime}$ are placed in the network of these two subsets, these nodes will not use any cooperative node values outside of themselves for updates. Consequently, the values of the entire system will remain unchanged, and the system will be unable to achieve consensus. Therefore, when the multi-agent system achieves asymptotic consensus, the system *G* is at least (S + 1)-robust.

#### (Sufficient Condition) proof

For $t \geqslant 0$, let ${\psi ^{\prime}_a}(t)$ be the clustering center of the system nodes at time t. In other words, it is proven that according to the above hybrid clustering review strategy, there exist nodes such that $\left| {x_{i}^{{}}\left( k \right) - {{\psi ^{\prime}}_a}(t)} \right| \leqslant \theta ^{\prime}$, thereby iteratively achieving system asymptotic consensus.

For discrete-time dynamic time nodes, when ${t^{}} \to \infty$, $t \in [k,k+1)$, and for $k \in Z$, ${\upsilon _i} \in {\text{C}} \cap {\nu ^D}$, according to Theorem 1, and ${\psi ^{\prime}_a}(t)$ is a bounded function with respect to t. Define $\Psi _{a}^{\prime }=\mathop {\lim }\limits_{{t \to \infty }} \psi _{a}^{\prime }(t)$, and for Formula (7), according to the S-local bounded model and the hybrid clustering review strategy, since the system is at least (2 S + 1) - robust, i.e., since there is at least one node in the (2 S + 1) - reachable set, implying the existence of $\left| {x_{i}^{{}}\left( k \right) - {{\Psi ^{\prime}}_a}} \right| \leqslant \theta ^{\prime}$, ${\Psi ^{\prime}_a} - \theta ^{\prime} \leqslant x_{i}^{{}}\left( k \right) \leqslant {\Psi ^{\prime}_a}+\theta ^{\prime}$. Similarly, ${\Psi ^{\prime}_a} - \theta ^{\prime} \leqslant x_{j}^{{}}\left( k \right) \leqslant {\Psi ^{\prime}_a}+\theta ^{\prime}$ holds, i.e., $\mathop {\lim }\limits_{{t \to \infty }} \left| {{x_i}\left( k \right) - {x_j}\left( k \right)} \right| \leqslant 2\theta ^{\prime}$. Let $\theta ^{\prime}=\frac{\varepsilon }{2}$; i.e., $\mathop {\lim }\limits_{{t \to \infty }} \left| {{x_i}\left( k \right) - {x_j}\left( k \right)} \right| \leqslant \varepsilon$. Thus, the asymptotic consensus of the hybrid multi-agent system is proven.

For continuous-time dynamic time nodes, when $t>{t^{\prime}}>0$, ${\upsilon _i} \in {\text{C}} \cap {\nu ^C}$, let $x_{i}^{{iM}}\left( {t^{\prime}} \right)$ be the maximum value at time *t*′. Suppose that the system cannot achieve asymptotic consensus, i.e., that for all cooperative nodes, $\left| {x_{j}^{i}\left( t \right) - x_{i}^{{}}\left( {t^{\prime}} \right)} \right|>\theta ^{\prime}$, i.e., $\forall {\upsilon _j} \in \left[ {\left( {{N_i} \cup \left\{ {{\upsilon _i}} \right\}} \right)\backslash {{\text{R}}_i}(t)} \right] \cap {\nu ^C}$, $\sum\limits_{{{\upsilon _j}}} {{\gamma _{ij}}\left( {x_{j}^{i}\left( t \right),x_{i}^{{}}\left( {t^{\prime}} \right)} \right)}>0$ holds; and $\forall {\upsilon _j} \in \left[ {{N_i}\backslash {{\text{R}}_i}(t)} \right] \cap {\nu ^D}$, $\sum\limits_{{{\upsilon _j}}} {{\gamma _{ij}}\left( {x_{j}^{i}\left( k \right),x_{i}^{{}}\left( t \right)} \right)}>0$ holds; thus, we obtain: ${\dot {x}_i}(t^{\prime})=\sum\limits_{{{\upsilon _j}}} {{\gamma _{ij}}\left( {x_{j}^{i}\left( t \right),x_{i}^{{}}\left( {t^{\prime}} \right)} \right)} +\sum\limits_{{{\upsilon _j}}} {{\gamma _{ij}}\left( {x_{j}^{i}\left( k \right),\;x_{i}^{{}}\left( t \right)} \right)}>0$. This finding contradicts the process of proving Theorem 1, specifically ${\dot {x}_i}(t^{\prime})=\sum\limits_{{{\upsilon _j}}} {{\gamma _{ij}}\left( {x_{j}^{i}\left( t \right),x_{i}^{{}}\left( {t^{\prime}} \right)} \right)} +\sum\limits_{{{\upsilon _j}}} {{\gamma _{ij}}\left( {x_{j}^{i}\left( k \right),x_{i}^{{}}\left( t \right)} \right)} =0$. Therefore, it is proven that the system can achieve asymptotic consensus.

#### Note

The above hybrid clustering review strategy includes both discrete-time system nodes and continuous-time system nodes under network attacks, and the system can achieve asymptotic consensus when it reaches (S + 1)-robustness.

#### Definition 2

(Asymptotic Proportional Consensus in Hybrid Multi-Agent Systems): In a hybrid multi-agent system, for any node’s initial state, over time, if all cooperative nodes can reach a steady state of consensus in a certain proportion, it is referred to as asymptotic proportional consensus in the hybrid multi-agent system. Specifically, given $({\lambda _1}, \ldots {\lambda _n})$, $\exists {\left\{ {x_{i}^{{}}\left( 0 \right)} \right\}_{{\upsilon _i} \in \nu }}$, $\varepsilon \to 0$, and $\forall {\upsilon _i},{\upsilon _j} \in {\text{C}} \cap {\nu ^C}$, there exists ${\lim _{t \to \infty }}\left| {{\lambda _i}{x_i}\left( t \right) - {\lambda _j}{x_j}\left( t \right)} \right| \leqslant \varepsilon$, and $\forall {\upsilon _i},{\upsilon _j} \in \text{C}$, there exists ${\lim _{k \to \infty }}\left| {{\lambda _i}{x_i}\left( k \right) - {\lambda _j}{x_j}\left( k \right)} \right| \leqslant \varepsilon$, then it is stated that the cooperative nodes ${\upsilon _i},{\upsilon _j} \in {\text{C}}$ of the hybrid multi-agent system achieve asymptotic proportional consensus.

Proportional consensus has many applications in daily life, such as in the control of multiple unmanned boat formations. In such cases, when the hybrid clustering review strategy mentioned above is applied, Eqs. (5), (6), and (7) can be modified as follows:

9 $${x^{\prime}_i}\left( t \right)=\operatorname{sgn} ({\lambda _i})\frac{1}{{\left| {\left\{ {x_{j}^{i}\left( t \right)} \right\}} \right|}}\sum\limits_{{j \in {\nu ^C} \cup {\nu ^D}}} {{\lambda _j}x_{j}^{i}\left( t \right)}$$

10 $${\dot {x}_i}(t)=\operatorname{sgn} ({\lambda _i})\sum\limits_{{{\upsilon _j}}} {{\phi _{ij}}\left( {{\lambda _j}x_{j}^{i}\left( t \right),{\lambda _i}x_{i}^{{}}\left( t \right)} \right)} +\sum\limits_{{{\upsilon _j}}} {{\phi _{ij}}\left( {{\lambda _j}x_{j}^{i}\left( k \right),{\lambda _i}x_{i}^{{}}\left( t \right)} \right)}$$

11 $${x_i}(k+1)=\operatorname{sgn} ({\lambda _i})\sum\limits_{{{\upsilon _j}}} {{\omega _{ij}}} (k){\lambda _j}x_{j}^{i}(k)$$

where, $\operatorname{sgn} ( \cdot )$ is the sign function, the function ${\phi _{ij}}$ needs to satisfy the three rules of Eq. (6), the clustering center discriminant formula is $\left| {{\lambda _j}x_{j}^{i}\left( t \right) - {{x^{\prime}}_i}\left( t \right)} \right| \leqslant \theta ^{\prime}$, and${\omega _{ij}}(k)$ needs to satisfy the first rule of Eq. (7) and $\sum\limits_{{{\upsilon _j} \in \left( {{N_i} \cup \left\{ {{\upsilon _i}} \right\}} \right)\backslash {{\text{R}}_i}(k)}} {\left| {{\lambda _j}} \right|{\omega _{ij}}(k)=1}$.

For the asymptotic proportional consensus of hybrid multi-agent systems, Theorem 1 and Theorem 2 are equally applicable.Specifically, when ${\lambda _1}={\lambda _2}= \cdots ={\lambda _n}=1$, Definition 3 is an extension of Definition 1.

## Experiments

On the basis of the research content of this paper, the following verification work was carried out: a hybrid multi-agent system with continuous time and discrete time (as shown in Fig. 2). Due to malicious network attacks, Byzantine nodes may exist in the system, causing task failure. Therefore, it is necessary to consider such situations so that the system can still achieve asymptotic consistency, that is, under the *S*-locally bounded model, the sufficient condition for the multi-agent system to achieve progressive consensus is that the hybrid multi-agent system is at least (2*S* + 1) -robust, and the necessary condition is that the hybrid multi-agent system is at least (*S*+ 1) -robust.

On the basis of the above, three simulation experiments are designed: Experiment 1 verifies the control effect of the model and strategy proposed in this paper on the hybrid multi-agent system with discrete time Byzantine nodes. Experiment 2 verifies the control effect of the proposed model and strategy on the continuous-time Byzantine nodes in the hybrid multi-agent system. Experiment 3 verifies the control effect of the model and strategy in this paper on the hybrid multi-agent system with both continuous time and discrete time Byzantine nodes.

**Fig. 3:**
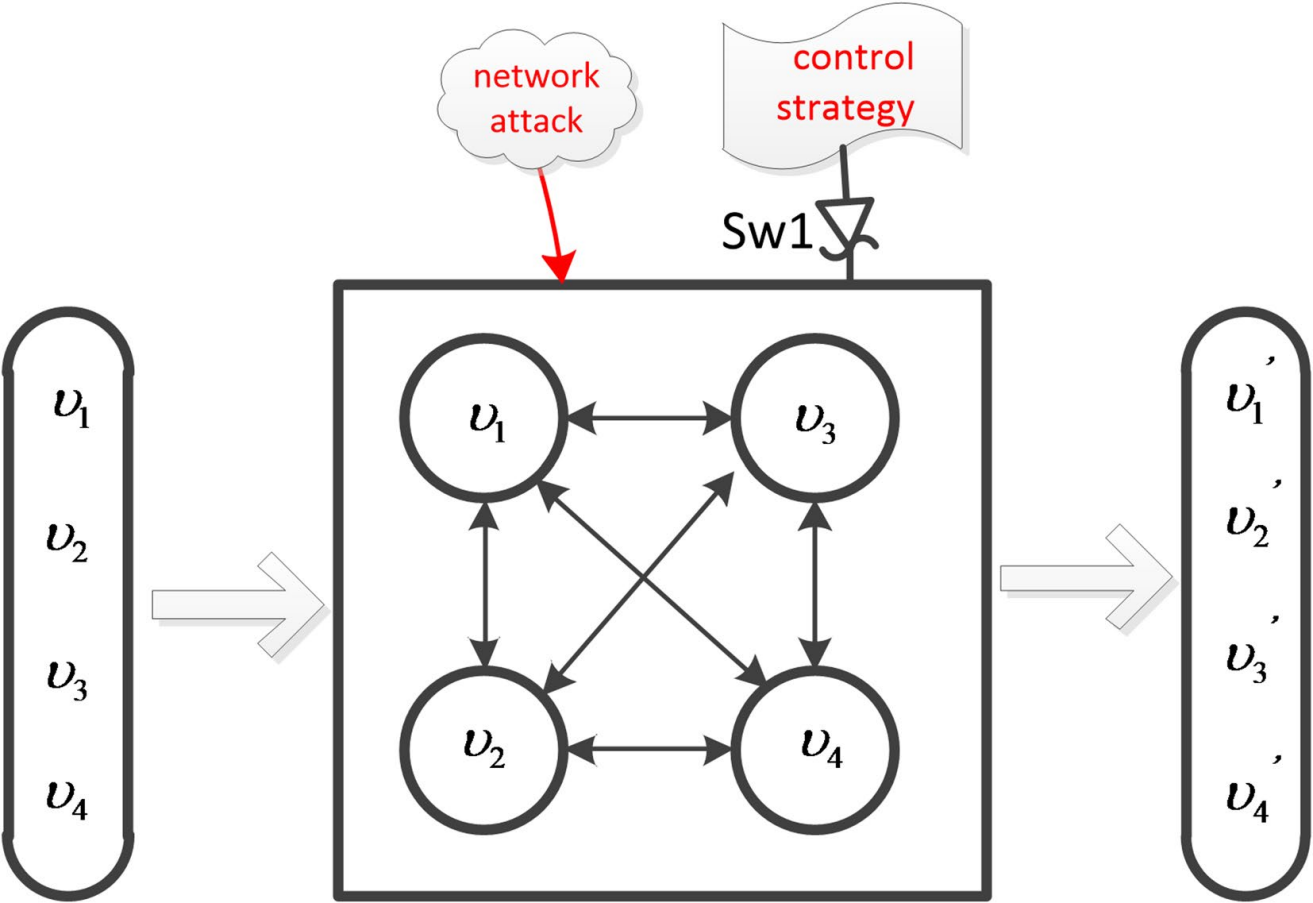
G is a 2-robust network system.

Considering the hybrid multi-agent system $G=(\upsilon ,{\varepsilon _{r(t)}},{H_{r(t)}})$ as shown in Fig. 3. where,${\nu ^C}=\left\{ {{\upsilon _1},{\upsilon _2}} \right\}$, ${\nu ^D}=\left\{ {{\upsilon _3},{\upsilon _4}} \right\}$, it is a 2-robust system, $v=\left[ {{\upsilon _1},{\upsilon _2},{\upsilon _3},{\upsilon _4}} \right]$ is the initial value, and $v^{\prime}=\left[ {{{\upsilon ^{\prime}}_1},\;{{\upsilon ^{\prime}}_2},\;{{\upsilon ^{\prime}}_3},\;{{\upsilon ^{\prime}}_4}} \right]$ is the operation result. $Sw1$ is the control switch, which controls whether to enable network attack or hybrid clustering control strategy.

**Fig. 4:**
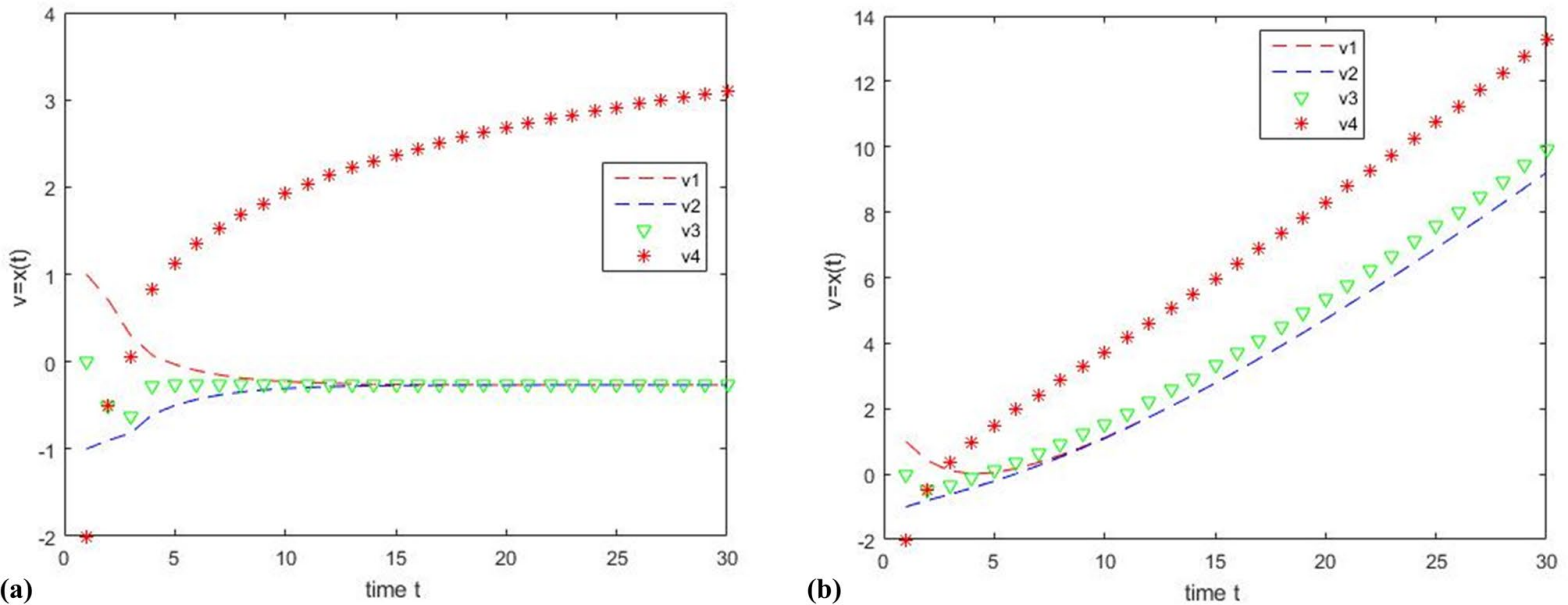
Comparison of results between using strategy algorithm and not using strategy algorithm when considering network attacks, where the Byzantine node is the discrete time node ${\text{B}}=\left[ {{\upsilon _4}} \right]$.

Experiment 1: Set the initial value as $v=\left[ {1, - 1,0, - 2} \right]$, set the threshold $\theta ^{\prime}=0.8$, and iterate 30 times. Let the cooperative node be ${\text{C}}=\left[ {{\upsilon _1},{\upsilon _2},{\upsilon _3}} \right]$, and let the Byzantine node be the discrete time node${\text{B}}=\left[ {{\upsilon _4}} \right]$, which takes the function ${x_i}(k+1)={x_i}(k)+\ln (k)$. Every continuous time node follows the function: ${\gamma _{ij}}\left( {x_{j}^{{}}\left( t \right),x_{i}^{{}}\left( t \right)} \right)=0.1*\left( {x_{j}^{{}}\left( t \right) - x_{i}^{{}}\left( t \right)} \right)$. Every discrete cooperative node adopts the function: ${\omega _{ij}}(k)={\left( {\left| {{N_i}} \right|+1 - \left| {{R_i}(k)} \right|} \right)^{ - 1}}$. The simulation results are shown in Fig. 4; Table 1.

**Table 1:** Comparison of simulation results between Fig. 4a, b.

Nodes	Initial Value	Function	Results of Fig. 4a	Results of Fig. 4b
${\upsilon _1}$	1	${\gamma _{ij}}\left( {x_{j}^{{}}\left( t \right),x_{i}^{{}}\left( t \right)} \right)$	−0.26	9.20
${\upsilon _2}$	−1	${\gamma _{ij}}\left( {x_{j}^{{}}\left( t \right),x_{i}^{{}}\left( t \right)} \right)$	−0.26	9.20
${\upsilon _3}$	0	${\omega _{ij}}(k)$	−0.26	9.92
${\upsilon _4}$	−2	${x_i}(k+1)={x_i}(k)+\ln (k)$	3.10	13.28

Figure 4a shows the simulation diagram using the hybrid clustering review strategy. Cooperative node, ${\text{C}}=\left[ {{\upsilon _1},{\upsilon _2},{\upsilon _3}} \right]$, can achieve asymptotic consensus and eliminate the influence of Byzantine nodes ${\text{B}}=\left[ {{\upsilon _4}} \right]$. Figure 4b shows the simulation diagram without the hybrid cluster review strategy. Under the influence of Byzantine nodes ${\upsilon _4}$, the cooperative nodes, ${\text{C}}=\left[ {{\upsilon _1},{\upsilon _2},{\upsilon _3}} \right]$, deviate from the center point and cannot reach the stable state; i.e., the asymptotic consensus cannot be achieved.

Table 1 compares the simulation results of Fig. 4a, b. This finding indicates that under the same conditions, the cooperative node ${\text{C}}=\left[ {{\upsilon _1},{\upsilon _2},{\upsilon _3}} \right]$ can achieve asymptotic consensus with the hybrid clustering review strategy in Fig. 4a, while the cooperative node, ${\text{C}}=\left[ {{\upsilon _1},{\upsilon _2},{\upsilon _3}} \right]$, cannot achieve asymptotic consensus without the hybrid clustering review strategy in Fig. 4b.

**Fig. 5:**
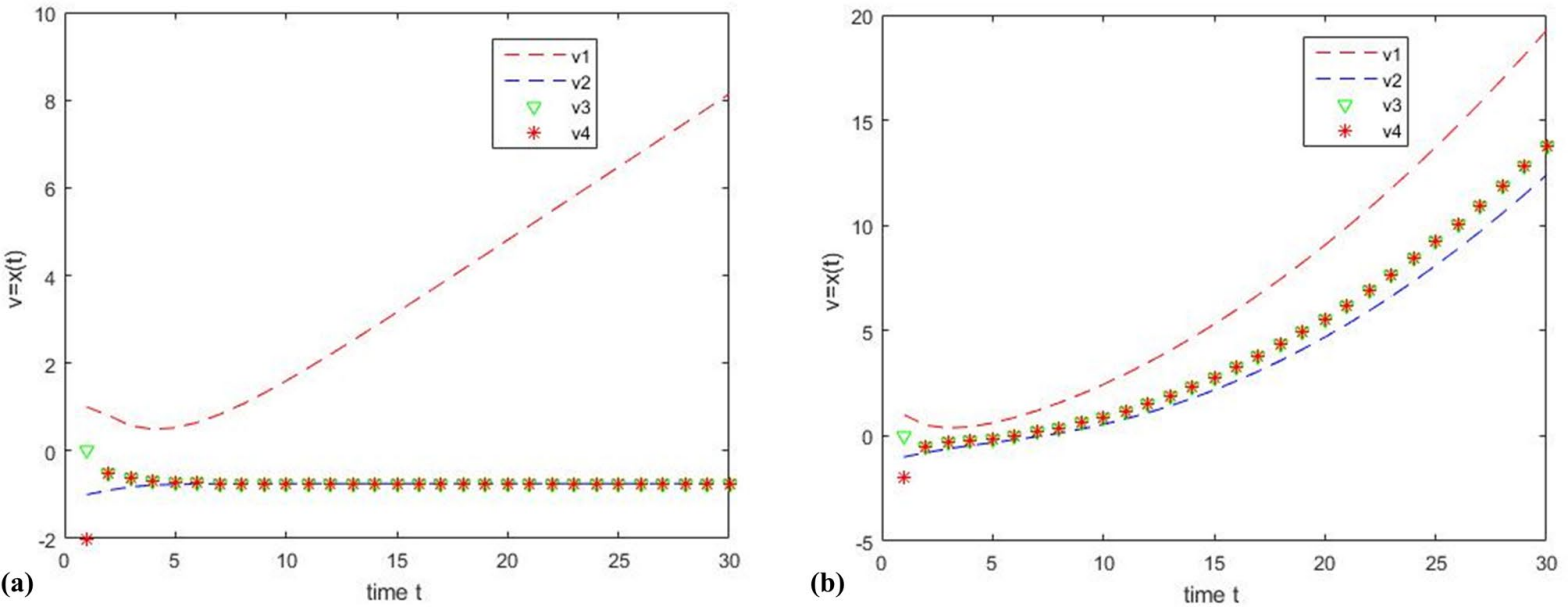
Comparison of results between using strategy algorithm and not using strategy algorithm when considering network attacks, where the Byzantine node is the continuous time node ${\text{B}}=\left[ {{\upsilon _1}} \right]$.

Experiment 2: Set the initial value as$v=\left[ {1,-1,0,-2} \right]$, set the threshold$\theta ^{\prime}=0.8$, and iterate 30 times. In this experiment, let the cooperative node be $\text{C}=\left[ {{\upsilon _2},{\upsilon _3},{\upsilon _4}} \right]$, and let the Byzantine node be the continuous time node ${\text{B}}=\left[ {{\upsilon _1}} \right]$, which follows the function :${\dot {x}_i}(t)={x_i}(t)+t/10$. The cooperative nodes C adopt the same protocol as in experiment 1. The simulation results are shown in Fig. 5; Table 2.

**Table 2:** Comparison of simulation results between Fig. 5a, b.

Nodes	Initial Value	Function	Results of Fig. 5a	Results of Fig. 5b
${\upsilon _1}$	1	${\dot {x}_i}(t)={x_i}(t)+t/10$	8.13	19.27
${\upsilon _2}$	−1	${\gamma _{ij}}\left( {x_{j}^{{}}\left( t \right),x_{i}^{{}}\left( t \right)} \right)$	−0.75	12.39
${\upsilon _3}$	0	${\omega _{ij}}(k)$	−0.75	13.79
${\upsilon _4}$	−2	${\omega _{ij}}(k)$	−0.75	13.79

Figure 5a shows the simulation diagram using the hybrid cluster review strategy, the cooperative node ${\text{C}}=\left[ {{\upsilon _2},{\upsilon _3},{\upsilon _4}} \right]$ can achieve asymptotic consensus and eliminate the influence of the Byzantine node ${\upsilon _1}$. Figure 5b shows the simulation diagram without the hybrid cluster review strategy. Under the influence of Byzantine nodes ${\upsilon _1}$, the cooperative nodes, ${\text{C}}=\left[ {{\upsilon _2},{\upsilon _3},{\upsilon _4}} \right]$, cannot reach the stable state; i.e., the asymptotic consensus cannot be achieved.

Table 2 compares the simulation results of Fig. 5a, b. This finding indicates that under the same conditions, the cooperative node ${\text{C}}=\left[ {{\upsilon _2},{\upsilon _3},{\upsilon _4}} \right]$ can achieve asymptotic consensus with the hybrid clustering review strategy in Fig. 5a, while the cooperative node ${\text{C}}=\left[ {{\upsilon _2},{\upsilon _3},{\upsilon _4}} \right]$ cannot achieve asymptotic consensus without the hybrid clustering review strategy in Fig. 5b.

**Fig. 6:**
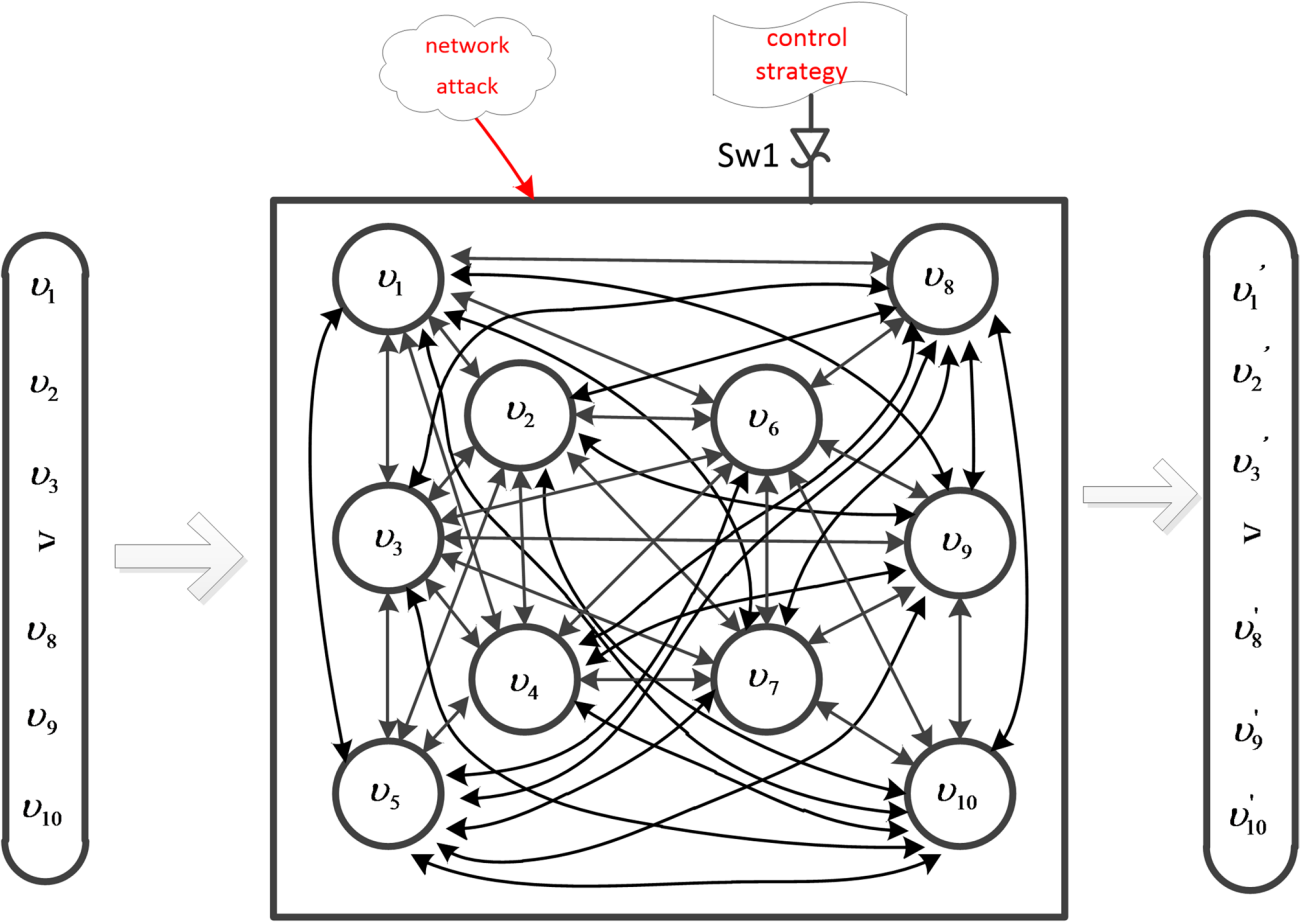
G is a 5-robust network system.

Consider a hybrid multi-agent system, $G=(\upsilon ,{\varepsilon _{r(t)}},{H_{r(t)}})$, with 10 nodes as shown in Fig. 6, where,${\nu ^C}=\left\{ {{\upsilon _1},{\upsilon _2},{\upsilon _{\text{3}}},{\upsilon _{\text{4}}},{\upsilon _{\text{5}}}} \right\}$, and ${\nu ^D}=\left\{ {{\upsilon _{\text{6}}},{\upsilon _{\text{7}}},{\upsilon _{\text{8}}},{\upsilon _{\text{9}}},{\upsilon _{{\text{10}}}}} \right\}$, it is a 5-robust system, $v=\left[ {{\upsilon _1},{\upsilon _2}, \ldots ,{\upsilon _9},{\upsilon _{10}}} \right]$is the initial value, and $v^{\prime}=\left[ {{{\upsilon ^{\prime}}_1},{{\upsilon ^{\prime}}_2}, \ldots {{\upsilon ^{\prime}}_9},{{\upsilon ^{\prime}}_{10}}} \right]$is the operation result. $Sw1$ is the control switch, which controls whether to turn on the network attack or the hybrid clustering control strategy.

**Fig. 7:**
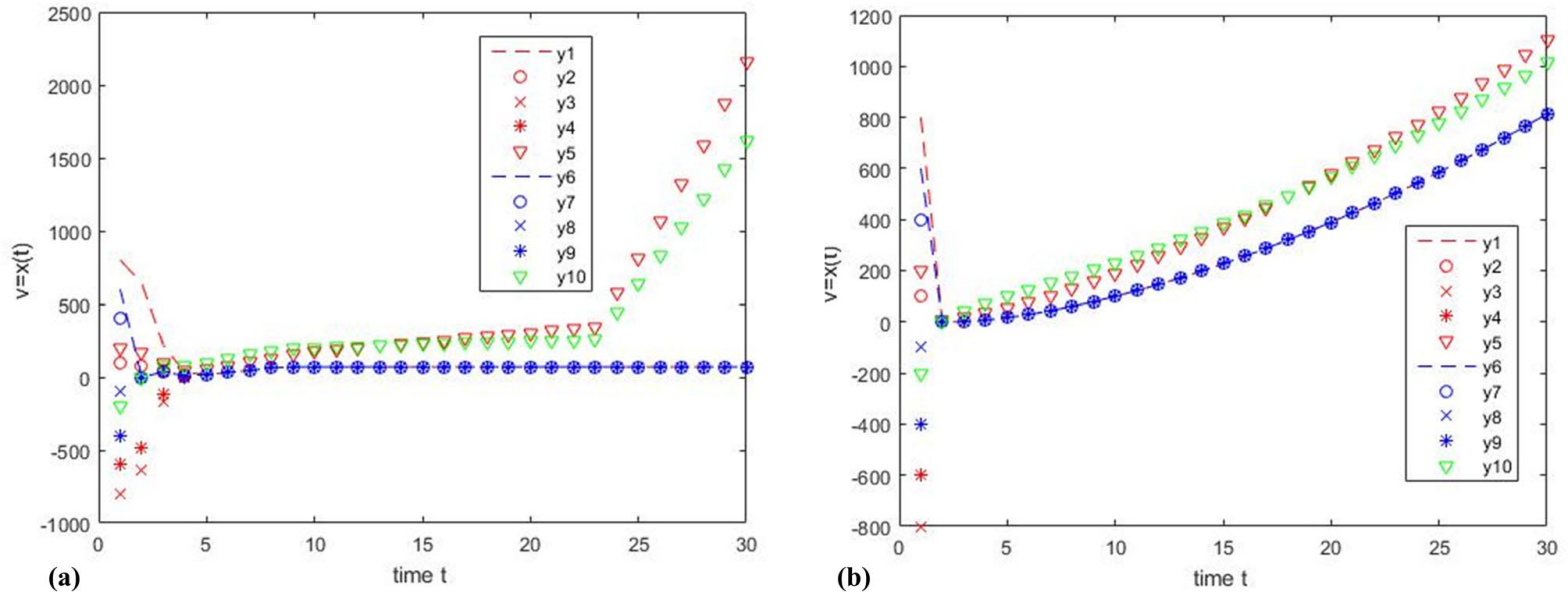
Comparison of results between using strategy algorithm and not using strategy algorithm when considering network attacks, where the Byzantine node are the node ${\text{B}}=\left[ {{\upsilon _5},{\upsilon _{10}}} \right]$, ${\upsilon _5}$ is the continuous time node, and ${\upsilon _{10}}$ is the discrete time node.

Experiment 3: Set the initial value *v* =[800 100–800 −600 200 600 400 − 100 −400 −200], set the threshold value $\theta ^{\prime}=200$, and the threshold value gradually decreases to 10, and the number of iterations is 30. In this experiment, let the cooperative node be ${\text{C}}=\left[ {{\upsilon _1},{\upsilon _2},{\upsilon _3},{\upsilon _4},{\upsilon _6},{\upsilon _7},{\upsilon _8},{\upsilon _9}} \right]$, and let the Byzantine node be ${\text{B}}=\left[ {{\upsilon _5},{\upsilon _{10}}} \right]$, and they follow the function separately: ${\upsilon _5}:{\dot {x}_i}(t)={\dot {x}_i}(t)+10{\text{*}}t$, and ${\upsilon _{10}}:{x_{10}}(k+1)={x_{10}}(k)+60*\ln (k)$, and the cooperation nodes C adopt the same protocol in experiment 1. The simulation results are shown in Fig. 7; Table 3.

**Table 3:** Comparison of simulation results between Fig. 7a, b.

Nodes	Initial Value	Function	Results of Fig. 7a	Results of Fig. 7b
${\upsilon _1}$	800	${\gamma _{ij}}\left( {x_{j}^{{}}\left( t \right),x_{i}^{{}}\left( t \right)} \right)$	69.8	813
${\upsilon _2}$	100	${\gamma _{ij}}\left( {x_{j}^{{}}\left( t \right),x_{i}^{{}}\left( t \right)} \right)$	69.8	813
${\upsilon _3}$	−800	${\gamma _{ij}}\left( {x_{j}^{{}}\left( t \right),x_{i}^{{}}\left( t \right)} \right)$	69.8	813
${\upsilon _4}$	−600	${\gamma _{ij}}\left( {x_{j}^{{}}\left( t \right),x_{i}^{{}}\left( t \right)} \right)$	69.8	813
${\upsilon _5}$	200	${\dot {x}_i}(t)={x_i}(t)+10*t$	216.2	1103
${\upsilon _6}$	600	${\omega _{ij}}(k)$	69.8	813
${\upsilon _7}$	400	${\omega _{ij}}(k)$	69.8	813
${\upsilon _8}$	−100	${\omega _{ij}}(k)$	69.8	813
${\upsilon _9}$	−400	${\omega _{ij}}(k)$	69.8	813
${\upsilon _{10}}$	−200	${x_{10}}(k+1)={x_{10}}(k)+60*\ln (k)$	162.2	1015

Figure 7a shows the simulation diagram using the hybrid clustering review strategy. Cooperative node ${\text{C}}=\left[ {{\upsilon _1}, \ldots ,{\upsilon _4},{\upsilon _6}, \ldots ,{\upsilon _9}} \right]$ can achieve asymptotic consensus and eliminate the influence of Byzantine nodes ${\text{B}}=\left[ {{\upsilon _5},{\upsilon _{10}}} \right]$. Figure 7b shows the simulation diagram without the hybrid clustering review strategy. Under the influence of Byzantine nodes, the cooperative nodes ${\text{C}}=\left[ {{\upsilon _1}, \ldots ,{\upsilon _4},{\upsilon _6}, \ldots ,{\upsilon _9}} \right]$ deviate from the center point and cannot reach the stable state, i.e., the asymptotic consensus cannot be achieved.

Table 3 compares the simulation results of Fig. 7a, b. This finding indicates that under the same conditions, the cooperative node ${\text{C}}=\left[ {{\upsilon _1}, \ldots ,{\upsilon _4},{\upsilon _6}, \ldots ,{\upsilon _9}} \right]$ can achieve asymptotic consensus with the hybrid clustering review strategy in Fig. 7a, while the cooperative node ${\text{C}}=\left[ {{\upsilon _1}, \ldots ,{\upsilon _4},{\upsilon _6}, \ldots ,{\upsilon _9}} \right]$ cannot achieve asymptotic consensus without the hybrid clustering review strategy in Fig. 7b.

By observing the above experiments (Figs. 3 and 6), Fig. 3 shows that the hybrid multi-agent system consists of 4 nodes and Fig. 6 shows that the hybrid multi-agent system consists of 10 nodes. Compared to static redundancy, the deep dynamic heterogeneous redundancy contains more functionally equivalent intelligent agents. By observing the above three sets of experiments (Fig. 4; Table 1; Fig. 5; Table 2, and Fig. 7; Table 3), Fig. 4; Table 1 shows that the system can achieve asymptotic consensus and eliminate the influence of Byzantine nodes of discrete time at t = 8. Figure 5; Table 2 shows that the system can achieve asymptotic consensus and eliminate the influence of Byzantine nodes of continuous time at t = 5. Figure 7; Table 3 shows that the system can achieve asymptotic consensus and eliminate the influence of Byzantine nodes of continuous and discrete time at t = 4. Compared to the W-MSR algorithm in references^12,16^, the clustering review strategy based on K-means in this paper can discover Byzantine points faster and achieve asymptotic consistency before t = 10. Compared to the dynamic heterogeneous redundancy architecture described in reference^28^, the deep dynamic heterogeneous redundancy architecture proposed in this paper introduces a forewarn strategy module based on asymptotic consistency. This strategy deeply simulates the state change process of each agent in the computation execution agent set, and can provide early warning of possible Byzantine nodes, thereby eliminating their influence.

## Conclusion

In this paper, the problem of active defense and asymptotic consensus control of hybrid multi-agent systems under network attack is studied. Considering the concepts of Byzantine nodes and active defense, a security cooperative control architecture model based on deep dynamic heterogeneous redundancy architecture (DDHR) is established. On the basis of Lyapunov’s concept of asymptotic stability, the definition of hybrid multi-agent asymptotic consensus is proposed. Furthermore, based on k-means clustering algorithm, a hybrid multi-agent asymptotic consensus clustering review strategy is proposed to solve the problem of hybrid multi-agent asymptotic consensus when Byzantine nodes exist. In addition, according to Lyapunov’s asymptotic stability theory, the sufficient conditions and necessary conditions for the system to achieve asymptotic consensus are proven, and the secure and rapid communication within the hybrid multi-agent is realized. Moreover, this conclusion is extended to the application of proportional asymptotic consensus. Based on the research foundation of this article, we will further investigate the solutions the surrounding control problem of multi-USVs under the false data injection (FDI) attacks.

## Data Availability

All the data generated or analysed during this study are included in this published article.
